# µ-Calpain Conversion of Antiapoptotic Bfl-1 (BCL2A1) into a Prodeath Factor Reveals Two Distinct alpha-Helices Inducing Mitochondria-Mediated Apoptosis

**DOI:** 10.1371/journal.pone.0038620

**Published:** 2012-06-20

**Authors:** Juan García Valero, Aurélie Cornut-Thibaut, Romain Jugé, Anne-Laure Debaud, Diana Giménez, Germain Gillet, Nathalie Bonnefoy-Bérard, Jesús Salgado, Gilles Salles, Abdel Aouacheria, Jérôme Kucharczak

**Affiliations:** 1 Institut de Biologie et Chimie des Protéines (IBCP), CNRS UMR 5086, Université Lyon 1, IFR 128, Lyon, France; 2 Laboratoire de Biologie Moléculaire de la Cellule (LBMC), Ecole Normale Supérieure de Lyon, CNRS UMR 5239, SFR Biosciences Gerland-Lyon Sud US8/UMS3444, Lyon, France; 3 Instituto de Ciencia Molecular, Universidad de Valencia, Paterna (Valencia), Spain; 4 Departamento de Bioquímica y Biología Molecular, Universidad de Valencia, Burjassot (Valencia), Spain; 5 Inserm U851, Université Lyon 1, IFR128, Lyon, France; 6 Centre de recherche en cancérologie de Lyon, Centre Léon Bérard, Université Lyon 1, Inserm U1052, UMS3453 CNRS, Lyon, France; Wayne State University School of Medicine, United States of America

## Abstract

Anti-apoptotic Bfl-1 and pro-apoptotic Bax, two members of the Bcl-2 family sharing a similar structural fold, are classically viewed as antagonist regulators of apoptosis. However, both proteins were reported to be death inducers following cleavage by the cysteine protease µ-calpain. Here we demonstrate that calpain-mediated cleavage of full-length Bfl-1 induces the release of C-terminal membrane active α-helices that are responsible for its conversion into a pro-apoptotic factor. A careful comparison of the different membrane-active regions present in the Bfl-1 truncated fragments with homologous domains of Bax show that helix α5, but not α6, of Bfl-1 induces cell death and cytochrome c release from purified mitochondria through a Bax/Bak-dependent mechanism. In contrast, both helices α5 and α6 of Bax permeabilize mitochondria regardless of the presence of Bax or Bak. Moreover, we provide evidence that the α9 helix of Bfl-1 promotes cytochrome c release and apoptosis through a unique membrane-destabilizing action whereas Bax-α9 does not display such activities. Hence, despite a common 3D-structure, C-terminal toxic domains present on Bfl-1 and Bax function in a dissimilar manner to permeabilize mitochondria and induce apoptosis. These findings provide insights for designing therapeutic approaches that could exploit the cleavage of endogenous Bcl-2 family proteins or the use of Bfl-1/Bax-derived peptides to promote tumor cell clearance.

## Introduction

Proteins of the Bcl-2 family are key regulators of mitochondrial outer membrane (MOM) permeabilization, a prerequisite to cytochrome c release from mitochondria and activation of the downstream apoptotic cascade that leads to cell demise [1], [2]. The Bcl-2 family comprises both anti-apoptotic (Bcl-2, Bcl-xL, Bcl-w, Bfl-1/A1 also named BCL2A1, Mcl-1 and Bcl2l10) and pro-apoptotic (e.g., Bax, Bak) members that share three BCL-2 homology (BH) motifs in their primary structure (BH1, BH2 and BH3), some anti-apoptotic members presenting an additional BH4 domain. Besides the multi-BH members, a class of proteins that contains a single BH3 domain, the so-called BH3-only proteins, also displays pro-apoptotic activity. Furthermore, many Bcl-2 family proteins contain a hydrophobic tail at their C-termini, called transmembrane (TM) domain, which may be critical for both subcellular localization and activity towards apoptosis. Mechanistically, death inducers like Bax and Bak are known to change conformation upon activation and oligomerize to form pores in the MOM, thus allowing the release of cytochrome c and other effectors of apoptosis from the mitochondria. In contrast, prosurvival members counteract the Bax- and Bak-induced MOM permeabilization, thus preserving the functional integrity of mitochondria, while BH3-only proteins provide an additional layer of complexity by activating either directly or indirectly Bax/Bak in response to noxious signals [3]. Astonishingly, despite their opposite effect on cell survival, both Bcl-2-like and Bax-like multidomain proteins share a common 3D globular structure in their water-soluble state, which also resembles that of some pore-forming bacteriocins, such as colicins and diphtheria toxin [4], [5]. By analogy with colicins, it was proposed that two central helices (α5-α6 in Bax, Bcl-2 and Bcl-xL and α6-α7 in Bid) within ‘globular’ Bcl-2 family members may participate in membrane insertion and pore formation, a model that was supported by the measurements of ion-channel activity in synthetic lipid membranes [6], [7], [8], [9]. Additional studies based on deletion mutants and site-directed mutagenesis underscored the crucial role of these central helices in the ion-channel activity, the release of cytochrome c and apoptosis regulation by both pro- and anti-apoptotic proteins [10], [11], [12], [13]. More recently, reductionist approaches showed that peptides corresponding to α5 and/or α6 of Bax can partly reproduce the poration activity displayed by the full-length protein in model membrane systems and mitochondria [14], [15], [16]. The pores appear to be of the mixed lipidic-peptidic type [14], [15], [17], [18], similar to those of membrane-active, amphipathic peptide antibiotics [19] and can be characterized as stable equilibrium structures [18], [20]. Interestingly, these studies have also pointed out differences in the respective abilities of these central helices to insert into model membranes or induce mitochondrial cytochrome c release depending on the parent protein from which they were derived (Bax, Bcl-xL or Bid), providing clues to the functional divergence observed within the family [14], [16], [21].

Besides regulating apoptosis through their mutual interactions, many Bcl-2-related proteins are known to undergo N-terminal protease-mediated truncation, a process that changes drastically their activity. Indeed, the prosurvival factors Bcl-2, Bcl-xL and Mcl-1 were shown to undergo caspase-dependent cleavage leading in all cases to the release of a pro-apoptotic truncated fragment [22], [23], [24], [25]. A different class of proteases named calpains has been reported to target both pro- and anti-apoptotic family members for proteolytic cleavage. Incidentally, µ-calpain has been shown to cleave Bax N-terminally [26] to release a truncated fragment presumed to behave like a BH3-only protein (similarly to the caspase cleavage products of Bcl-2, Bcl-xL and Mcl-1) [27]. Notably, we and others previously demonstrated that the same calpain converts prosurvival Bfl-1 into a death-promoting factor in B cells through N-terminal truncation [28], although in this case precise characterization of the cleavage sites/products is lacking.

In this paper, we have mapped biochemically the µ-calpain cleavage sites present on the Bfl-1 protein and identified the potential death-promoting fragments of Bfl-1 that are generated upon µ-calpain digestion. The MOM-permeabilizing and death-promoting abilities of the different membrane-active regions present in the Bfl-1 truncated fragments were compared to that of homologous domains in pro-apoptotic Bax using cell-based assays and isolated mitochondria. Our data indicate that, although both proteins are cleaved by the same protease, Bfl-1 being converted into a potent pro-apoptotic factor, their toxic domains have only limited overlap and induce apoptosis through dissimilar mechanisms. The significance of these findings for the potential applicability of Bax/Bfl-1-derived peptides as therapeutic agents is discussed.

## Results

### Bfl-1 is Cleaved at the N-terminus by µ-calpain in a way Similar to Bax

Both Bax and Bfl-1 undergo µ-calpain cleavage [26], [28]. However, the sites targeted by the protease and the nature of the subsequent cleaved fragments remained to be determined for Bfl-1.

**Figure 1:**
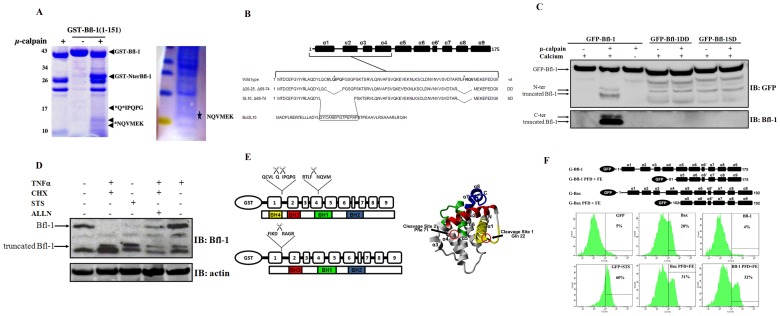
μ-calpain cleaves Bfl-1 at two major sites in its N-terminus and releases a large C-terminal fragment with cytotoxic activity. (A) GST-Bfl-1(1–151) was digested with recombinant µ-calpain *in vitro* and the fragments were separated by SDS-PAGE (left panel). Bands corresponding to cleaved products (arrows) were analyzed by mass spectrometry (MS). A higher concentration of recombinant GST-Bfl-1(1–151) was treated with µ-calpain, products were separated by SDS-PAGE and blotted to PVDF (right panel). A sub-band (asterisk) was excised and subjected to Edman degradation, which indicated that Bfl-1 had been N-terminally cleaved between residues F71 and N72. (B) Schematic representation of the wild type Bfl-1 protein showing the location of the identified μ-calpain cleavage sites (asterisks, upper sequence), of mutant Bfl-1 protein with a 6 aminoacids deletion surrounding the two cleaved residues (Bfl-1DD, middle sequence), and of mutant Bfl-1 in which the region overlapping the first cleavage site was swapped with a structurally homologous region in Bcl2L10 (Bfl-1SD, bottom sequence) (C) Confirmation of the two calpain cleavage sites identified in Bfl-1 using noncleavable mutants. 293T cell lysates expressing GFP-tagged Bfl-1 constructs were exogenously treated with μ-calpain. Lysates containing equal amount of GFP-tagged Bfl-1 proteins were separated by SDS-PAGE and analyzed by western blot with an anti-GFP antibody to detect the full length protein and N-terminal truncated fragments and with a polyclonal anti-Bfl-1 antibody to detect C-terminal truncated fragments. Upper and lower panels represent two independent experiments with different time of exposure. (D) BJAB cells were cultured with or without treatment with TNF/CHX in the presence or absence of the calpain inhibitor ALLN. Lysates were separated by SDS-PAGE and the presence of a cleaved fragment was assayed by western blot using an anti-Bfl-1 antibody. (E) Secondary structure of Bfl-1 in which the nine helices of the protein are represented by boxes along with the different BH domains (left panel).The two cleavage sites mapped in (A) are indicated. A comparison with the previously published µ-calpain cleavage site in Bax is also shown [26] (bottom sequence). 3D structure of Bfl-1 (2VM6) [31] and position of the different cleaved sites (red circles) are indicated. The different Bcl-2 Homology domains are colored in yellow (putative BH4), red (BH3), green (BH1) and blue (BH2). (F) FACS assays of Annexin V staining in HT1080 cells. Chimeric GFP constructs encoding GFP alone, or fusions of GFP with full-length Bfl-1 or Bax or with the various membrane-active α-helices corresponding to the C-terminal part of Bfl-1 or Bax, i.e. α5, α6 (PFD, pore forming domain) and α9 (FE, final exon) are represented. The α-helical topology of Bax and Bfl-1 corresponds to the structures solved in aqueous environment [31], [50]. Transfected cells were stained for phosphatidylserine exposure using Cy3-conjugated Annexin V and the percentage of apoptotic GFP-expressing cells was determined by FACS 24 hours post transfection (right panel). Death of GFP-expressing and staurosporine (STS)-treated cells were also monitored as controls. Graphs shown are representative of three independent experiments.

To characterize the precise cleavage sites in Bfl-1, GST-Bfl-1(1–151) recombinant protein was digested by µ-calpain and the produced fragments were separated by SDS-PAGE and analyzed by mass spectrometry (MS). A major band of ∼28,000 Da corresponding to the GST-containing N-terminal part of Bfl-1 was detected. In addition, two fragments of ∼12,000 Da and 17,500 Da were also found, suggesting that µ-calpain targets Bfl-1 on two major sites (Figure 1A, left panel). Samples corresponding to these bands were digested by trypsin and the resulting peptides were then analyzed by MS. Mass spectra reproducibly detected a cleavage after L21 and Q22 for the top sub-band and after F71 for the bottom sub-band. The residue Q22 was predicted with a high score as a cleavage site in Bfl-1 by the CaMPDB program [29]. This residue is located in a disordered linker, i.e. a type of sites that often corresponds to preferentially scissible regions [30]. To further support the calpain cleavage sites, sub-bands were sequenced using Edman degradation, which confirmed that Bfl-1 had been N-terminally cleaved between residues F71 and N72 (Figure 1A, right panel). Moreover, to exclude the possibility that additional cleavage sites are present downstream of the F71/N72 residues, full length GST-Bfl-1(1–172) and GST-Bfl-1(1–151) were treated with µ-calpain and products were separated by SDS-PAGE. Gels show that the size of the lower band corresponding to the cleaved C-terminal part of the protein shifts upwards when full length Bfl-1 is digested, therefore demonstrating that there is no additional cleavage site after the F71–N72 residues (Figure S1).

No single substrate-binding/cleavage site has been defined for calpains because these proteases display conformational preference, implying that the region surrounding the cleavage site is important for recognition by the protease. Thus, in order to test whether mutation of the identified cleavage sites blocks proteolysis by µ-calpain, we generated a double deletion mutant of Bfl-1 lacking residues 20–25 and 69–74 (Bfl-1DD). As deletion of the residues 20–25 occurs within the putative BH4 region of Bfl-1, a domain critical for the cytoprotective activity of antiapoptotic Bcl-2 proteins [31], [32], we designed an additional mutant (Bfl-1SD) in which the region surrounding the first cleavage site was swapped with a structurally homologous region present in BCL2L10, a close homologue of Bfl-1 that does not exhibit a calpain cleavage site according to the CaMPDB database [29] (Figure 1B, Figure S2). When lysates from 293T cells transiently expressing comparable levels of GFP-Bfl-1 or GFP-tagged Bfl-1 mutants were treated with recombinant calpain, the double bands corresponding to the N- or the C-terminal fragments of Bfl-1 were visible only for the case of the wild type protein but not for any of the two mutants (figure 1C). The transient expression of the Bfl-1DD and Bfl-1SD mutants appeared to induce cell death both in 293T and Hela cells (data not shown and Figure S3) thus precluding the establishment of stable cell lines to gain insights into the underlying mechanisms.

Nevertheless, as the μ-calpain-dependent cleavage of Bfl-1 was observed both *in vitro* and for ectopically expressed Bfl-1 in cells, we investigated whether such truncation of Bfl-1 could also be detected in cells with high level of endogenous Bfl-1. We used BJAB, a Diffuse Large B Cell lymphoma (DLBCL) cell line known to express high levels of Bfl-1 mRNA and protein [33], [34]. A treatment with TNFα and CHX or staurosporine (STS), two stimuli known to activate μ -calpain in B cells induced the appearance of a band at ∼12 kDa, in agreement with the size previously observed *in vitro* for the C-terminal truncated fragment of Bfl-1. Importantly, the appearance of the cleaved fragment was abrogated in presence of the calpain inhibitor ALLN (figure 1D), demonstrating that μ-calpain-mediated cleavage of Bfl-1 also occurs with the endogenous protein. Altogether, these results strongly suggest that Bfl-1 undergoes µ-calpain-mediated truncation at two major sites; the first one at the end of helix α1, similar to the cleavage in Bax, and the second one within helix α4 (before the BH1 domain) (Figure 1E).

As the BH3 domain of Bfl-1 is poorly conserved within the Bcl-2 family [31] and as the BH3 domain peptide of Bfl-1 displays no binding activity against the anti-apoptotic Bcl-2 proteins [35], we reasoned that it is unlikely that this region is involved in the cytotoxic effect of µ-calpain cleaved Bfl-1. Therefore, we focused on the membrane-active fragments present on the truncated C-terminal part of Bfl-1, as these regions were the most likely candidates for playing a role in cell death induction. At an early stage of the work, we generated GFP fusion constructs of Bfl-1 encompassing the C-terminal helices downstream the µ-calpain cleavage site, a region that covers the pore-forming domain (PFD) and the carboxy-terminal 35 amino acids encoded by the last exon (FE, final exon) (figure 1F, top panel). Cells transfected with GFP or GFP-Bfl-1 showed no apoptotic features while GFP-Bax and GFP-Bax-(PFD+FE) exhibited higher apoptotic rates. Unexpectedly, the GFP-Bfl-1 (PFD+FE) fusion protein that contains helices downstream of the µ-calpain cleavage site (but is devoid of any BH3 domain) induces apoptosis akin to the homologous miniature of Bax (figure 1F, bottom panels).

Overall, these results indicate that the larger truncated Bfl-1 fragment containing α5 to α9 helices is likely responsible for the cytotoxic effect of Bfl-1 observed upon µ-calpain-mediated cleavage.

### Bfl-1 α5/α9-containing Constructs Induce Apoptosis via Bax/Bak-dependent and Independent Mechanisms

**Figure 2:**
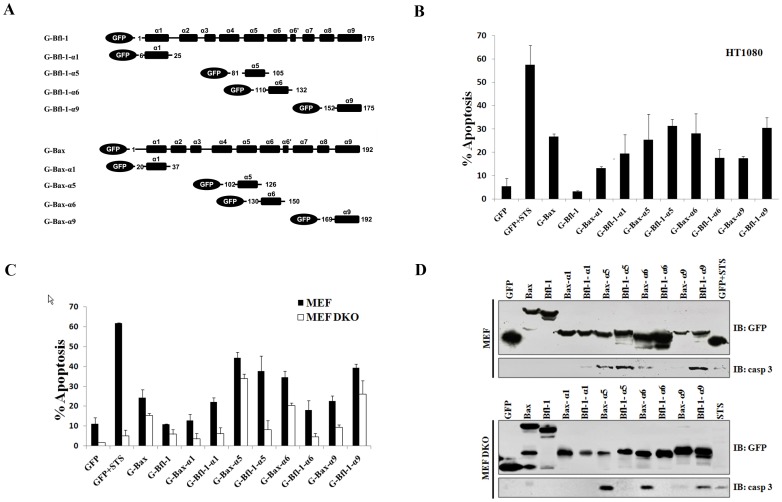
The ectopic overexpression of GFP-tagged Bfl-1-α5/α9 and Bax-α5/α6 fragments induces cell death but with distinct Bax/Bak requirements. (A) Chimeric GFP proteins used in this study. GFP-tagged constructs encoding GFP alone, or fusions of GFP with full-length Bfl-1 or Bax or with the various membrane-active α-helices of Bfl-1 or Bax, i.e. α1, α5, α6 and α9, are represented. The α-helical topology of Bax and Bfl-1 correspond to the structures solved in aqueous environment [31], [50]. Because the structures of the membrane-bound forms of these proteins are unknown, we designed sequence boundaries that extend a few residues beyond the α-helical regions in the structures of the water-soluble forms. (B) FACS assays of Annexin V staining in HT1080 cells. Transfected cells were stained for phosphatidylserine exposure using Cy3-conjugated Annexin V and the percentage of apoptotic GFP-expressing cells was determined by FACS. Histograms represent the percentage of GFP-expressing cells binding Annexin V (upper panel). Assays were performed in triplicate (error bars correspond to standard deviations). Staurosporine (STS) treatment was included for comparison. (C) FACS histogram showing Annexin-V staining of MEF (left panel) and MEF-DKO cells (right panel) expressing the different GFP-tagged constructs described in (A). GFP-transfected cells treated with staurosporine (STS) or left untreated were used as controls. Assays were performed in triplicate (error bars correspond to standard deviations). (D) Expression and analysis of the various GFP-tagged proteins in mammalian cells. Western Blot analyses on transiently transfected MEF (top panel) or MEF DKO cells (bottom panel) at 24 h post-transfection. Proteins were separated by SDS-PAGE and the presence of fusion proteins with the correct size was tested by immunoblot with anti-GFP antibody. Analysis of caspase-3 activation below each panel shows the cleaved 17 kDa product indicative of activated caspase-3.

To compare the contribution of each structural domain of Bfl-1 and Bax to apoptosis induction, the putative membrane-active α-helices of Bfl-1, as well as homologous helices within Bax, were cloned in fusion with GFP (Figure 2A). The different constructs were transfected into HT1080 cells and GFP-positive cells were analyzed by FACS following annexin V labeling. As expected, cells expressing GFP-Bax showed apoptotic features. The expression of the α5 helices from Bax and Bfl-1 both induced significant cell death, Bax-α6 having a similar toxic effect but not Bfl-1-α6. In addition, the C-terminal helices of both proteins showed markedly different effects. Indeed, Bax-α9 produced only weak apoptotic induction, while Bfl-1-α9 displayed a sharp increase in apoptotic rate (Figure 2B), which was comparable to that of Bfl-1-α5. From these results it is clear that both helices α5 and α9 of Bfl-1 have strong and independent cell death-inducing activities.

To further investigate the mechanisms by which the different constructs promote cell death, we used wild type MEF (WT) and bax/bak double knockout MEF (DKO) cells in parallel (figure 2C). Cell death quantification by FACS showed that Bfl-1-α5 induced cell death in WT but not in DKO cells. This pattern is different from that of Bax-α5 and Bfl-1-α9, since both have a potent toxic effect regardless of Bax/Bak expression. Similarly, α6 helices of Bfl-1 and Bax exhibited again strong differences, as Bfl-1-α6 was inefficient for inducing cell death while Bax-α6 was a potent cell death inducer in both cell types. To further evaluate the contribution of each helix of Bfl-1 and Bax for the induction of apoptosis, GFP-tagged constructs were transfected into either WT or DKO MEF and cell lysates were subsequently analyzed by immunoblotting. Samples expressing comparable levels of GFP-tagged proteins were assayed for caspase 3 activation as a marker of apoptosis (figure 2D). While Bax-α5 and Bax-α6 induced strong caspase 3 cleavage, regardless of the presence of Bax and Bak, Bfl-1-α5 induced caspase 3 activation only in WT MEFs. In contrast, Bfl-1-α6 failed to trigger caspase 3 activation in both cell lines. Finally, Bfl-1-α9 induced massive caspase 3 activation in both WT and DKO MEF cells, but Bax-α9 had no significant effect (Figure 2D). Taken together, these data demonstrate that individual membrane-active helices from Bfl-1 have different abilities to induce cell death when compared among each other (Bfl-1-α5, Bfl-1-α9>Bfl-1-α6, Bfl-1-α1) or with homologous fragments on Bax (Bax-α6>Bfl-1-α6; Bfl-1-α9>Bax-α9). The data also show that cell death induction by Bfl-1-α5 operates through a mechanism that depends on endogenous Bax/Bak, while the action of Bax-α5, Bax-α6 and Bfl-1-α9 is independent of the intrinsic expression of these proteins in the cultured cells.

To assess the contribution of each helix in the context of the whole C-terminal truncated part of Bfl-1, we expressed larger GFP-fused constructs that comprise α5, α6 and α9 helices of Bfl-1 in WT and DKO MEF cells. Interestingly, the α5-α6 or α5-α6-α9 GFP chimeras of Bfl-1 behaved like α5 alone as they showed a strong apoptotic effect and appeared to require Bax/Bak to induce cell death (Figure S4). These data suggest that although α9 of Bfl-1 has a toxic potency by itself, in fragments where the α9 helix is present together with α5, like in the C-terminal part of truncated Bfl-1, it is the latter helix that governs the mechanism of action that drives cells into apoptosis.

### Homologous Membrane-active Helices from Bfl-1 and Bax Exhibit Similar Subcellular Localization

**Figure 3:**
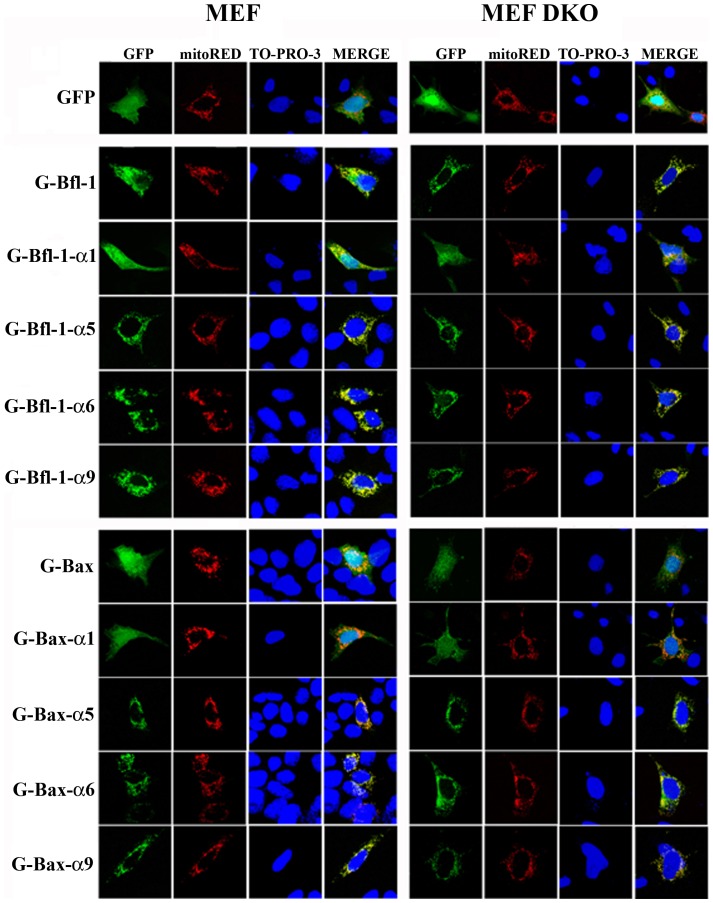
Subcellular localization of the GFP-tagged, Bfl-1/Bax-derived (poly)peptides. MEF (left panels) and MEF DKO (right panels) cells were co-transfected with mitoDsRed plasmid (encoding DsRed2 fused to the mitochondrial targeting sequence from subunit VIII of human cytochrome c oxidase) and the GFP-tagged constructs. Subcellular distribution was analyzed by confocal microscopy 24 h after transfection. Confocal images showing GFP (green) and MitoDsRed (red) fluorescence. The DNA staining dye Topro-3 (blue) was used to visualize the nuclei. In merged images, the yellow color shows the co-localization of GFP and MitoDsRed at mitochondria. Scale bar, 10 µm.

To study the mechanism by which membrane-active fragments derived from Bfl-1 and Bax proteins induce apoptosis, we compared the subcellular distribution of the different GFP-fused constructs by confocal fluorescence microscopy. Expression of the fusion proteins yielded abundant and intense GFP fluorescence in transfected WT or DKO cells (Figure 3). GFP alone showed a diffuse localization. In contrast, full length Bfl-1 as well as Bfl-1 helices exhibited a partial (α1) or a strong (α5, α6 and α9) clustered staining corresponding to MitoDsRed-labeled mitochondria. In parallel, the subcellular localization of the homologous helices of Bax and Bfl-1 was compared. We observed that Bax-α1 exhibited a weak mitochondrial staining similar to that of Bfl-1-α1. The other α-helices from Bax (α5, α6 and α9) targeted GFP to the mitochondrial membranes with the same strong clustered pattern observed for the corresponding Bfl-1 helices. However, the membrane active helices that had shown no toxic effect, i.e. Bfl-1-α1 and Bfl-1-α6, were addressed to mitochondria as were those that induced cell death. Therefore, although each helix of Bax and Bfl-1 shares a similar propensity to target mitochondria, additional intrinsic differences exist that explain the differential cytotoxicity of the helices. Importantly, the absence of endogenous Bax and Bak did not affect the subcellular localization of the various GFP constructs (figure 3), suggesting that the amphipathic characteristics of Bfl-1 or Bax membrane-active helices may be sufficient to target the fusion proteins to mitochondria independently of Bax and Bak. Taken together, these results show that the differential toxic effect observed between Bfl-1 helices is not due to differential localization of the expressed chimeric constructs.

### Synthetic Peptides Derived from Helices α5, α6 or α9 of Bfl-1 Induce Cytochrome C Release from Mitochondria with Different Efficacy and Mechanisms

**Figure 4:**
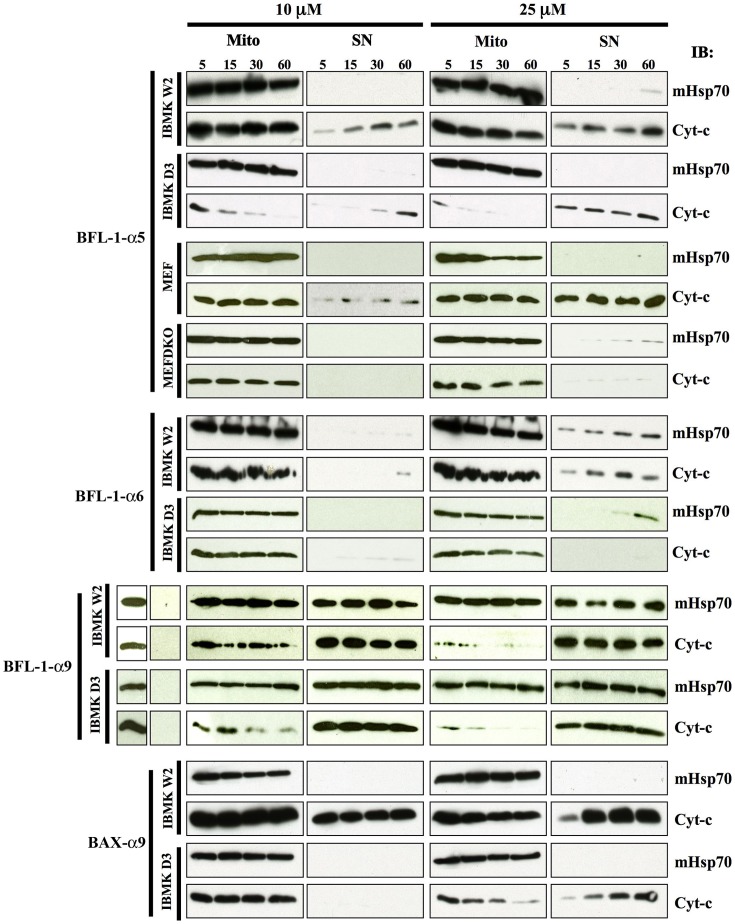
Bfl-1-derived peptides have different abilities to permeabilize the MOM of mitochondria isolated from cultured cells. Peptides were incubated at different concentrations (10 µM and 25 µM) with isolated mitochondria for the indicated times (5, 15, 30 and 60 min) and the release of cytochrome c was monitored by immunoblot (IB). MitoHsp70 (mHsp70) was used as control indicative of equal-loading and proper isolation of the pellet fraction containing mitochondria (Mito) in comparison to the supernatant fraction (SN). Cytochrome c release assays were performed using iBMKW2 (wild type) and iBMKD3 (double KO Bax/Bak) for all tested peptides. For Bfl-1-α5, wild type MEF and MEF DKO (Bax/Bak −/− double KO) cells were used in parallel.

To explore the effect of Bfl-1-derived fragments on mitochondrial membrane permeabilization, isolated mitochondria were used as a test system which closely resembles the *in cellulo* functional context. To this end, different synthetic peptides were directly incubated with mitochondria purified from both WT and DKO MEF or iBMK cells at different concentrations and times. We monitored the permeabilization of mitochondria by assaying the presence of cytochrome c in the pelleted fraction (mitochondria) and in the supernatant. In parallel, detection of the mitochondrial matrix-resident protein Hsp70 was used to validate the proper isolation of mitochondria. Among the peptides derived from Bfl-1 central helices, the Bfl-1-α5 peptide was able to release efficiently cytochrome c at a 10 µM concentration and 5 min incubation time on mitochondria from two different WT cells. Importantly, the release was significantly delayed when mitochondria from two types of Bax/Bak double KO cells were used, indicating that poration of the mitochondria upon Bfl-1-α5 treatment is modulated by endogenous Bax and/or Bak proteins (figure 4, upper panels). In contrast, the Bfl-1-α6 peptide failed to induce cytochrome c release in these experiments, even when high peptide concentrations and incubation times were used (figure 4, middle panels).

Next, our *in vitro* assays showed that cytochrome c release occurred massively at low concentrations when a Bfl-1-α9 peptide was used and that mitochondria were totally depleted of cytochrome c at 25 µM even at short times of peptide exposure. Unexpectedly, the presence of the Hsp70 protein was detected in both the mitochondrial and the supernatant fractions at all three peptide concentrations. In parallel, the same mitochondria extracts treated with DMSO for 60 minutes revealed that both Hsp70 and cytochrome c were in the pellet, ruling out that a contamination occurred between the mitochondria and the supernatant fractions during the experimental procedure (figure 4, lower panels). Importantly, when a Bax-α9 peptide was applied to mitochondria, concentration-dependent cytochrome c release was observed in WT cells but not in DKO cells although the HSP70 marker was properly distributed into the pellet fraction. These results indicate that Bfl-1-α9 uses a distinct mechanism to porate mitochondria compared to Bfl-1-α9.

**Figure 5:**
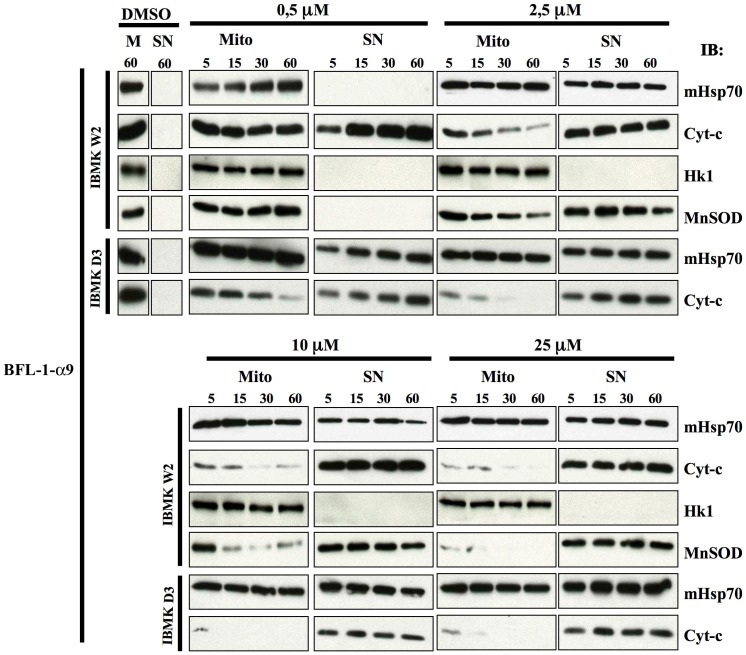
The Bfl-1-α9 peptide induces mitochondrial permeabilization through a membrane-destabilizing mechanism. Bfl-1-α9 peptide was incubated for the indicated times with mitochondria at lower concentrations (0.5 µM and 2.5 µM, top panels) and previously used in figure 4 (10 µM and 25 µM, bottom panels). The release of cytochrome c and the expression of MitoHsp70 were monitored as in figure 4, combined with the detection of the external membrane associated protein hexokinase 1 (HK1) and the matrix-contained protein MnSOD.

To investigate further the mechanism by which Bfl-1-α9 permeabilizes mitochondrial membranes, we assayed the distribution of MnSOD, another matrix-resident protein (like Hsp70), and of Hexokinase 1 (HK1), an integral outer membrane protein. After treatment with Bfl-1-α9 at concentrations in the range 2.5–25 µM, MnSOD was found both in the pelleted and supernatant fractions, in a way similar to Hsp70. In sharp contrast, the membrane resident protein HK1 was restricted to the pellet (figure 5). These results indicate that up to 2.5 µM concentration Bfl-1-α9 was able to porate both the outer and inner mitochondrial membranes. However, it does not appear to exert an extensive disruption action (detergent-like action [36] over membranes since HK1 was not solubilized. Notably, at 0.5 µM concentration, the peptide was still able to induce a strong release of cytochrome c even at short incubation times (figure 5), but the integrity of the mitochondrial inner membranes was somehow preserved as Hsp70 was only found in the pellet in that case. Taken together, these results show that Bfl-1-α9 has a high potency to permeabilize mitochondrial membranes, as demonstrated by the induced leakage of high molecular weight proteins such as MnSOD or Hsp70 from the mitochondrial matrix to the cytosol. Moreover, this peptide behaves differentially to porate the MOM depending on the concentration used.

## Discussion

The mechanism by which pro- and anti-apoptotic members of the Bcl-2 family operate within the MOM governs the leaking of apoptogenic factors from the mitochondria and finally the ‘live-or-die’ fate of the cell. Interestingly, despite opposite effects on apoptosis and wide differences in amino acid sequences, ‘multi-BH’ Bcl-2 family proteins share a similar helical bundle fold that resembles the pore-forming domains of some bacterial toxins [37], [38]. Although many 3D structures of multidomain Bcl-2 proteins are now resolved [39], the structural determinants that are key elements to the functional differences between pro- and anti-apoptotic members remain largely unknown. In this paper, we have compared the various membrane-active domains of anti-apoptotic Bfl-1 with their homologous helices found in pro-apoptotic Bax with respect to their ability to induce cytochrome c release from isolated mitochondria, to target GFP to mitochondrial membranes and to induce cell death. Our results clearly show that structural domains distinct from the BH3 are able to induce cell death and therefore represent an alternative way for cleaved Bcl-2-related proteins to fulfill their cytotoxic effect. In addition, extensive comparison of these domains shows that homologous regions behave differently in their ability to induce mitochondrial permeabilization and have different molecular requirements (i.e. presence of endogenous Bax or Bak) for inducing apoptosis depending on whether they derive from Bfl-1 or Bax.

A striking example comes from our findings that helix α6 of Bfl-1 is unable to induce cell death and to release cytochrome c whereas the corresponding helix on Bax has strong cytotoxic properties [40], [41]. Consistent with this, we have previously reported that helix α6 of anti-apoptotic Bcl-xL does not insert efficiently into purified microsomal membranes [21] and is not endowed with the capacity to induce cytochrome c release from purified mitochondria [16] contrary to the equivalent helix in Bax. In contrast, we found that helices α5 from both Bax [16], [41] and Bfl-1 (this study) were individually sufficient to induce cell death. However, quite unexpectedly, Bfl-1-α5 (but not Bax-α5) [41] appeared to require Bax/Bak for both its cytochrome c release and death-inducing activities. The origin of this different behavior is not clear but may be related to the fact that, while Bax-α5 has been shown to form Bax-like pores in model membranes [14], [17], [18], full length Bfl-1 was reported to selectively interact with endogenous Bak (but not Bax) to suppress apoptosis [42]. This observation raises the possibility that Bfl-1-α5 could have the capacity to stimulate the membrane-permeabilizing function of Bak through direct physical contact. Alternatively, Bfl-1-α5 may be responsible for functional Bax or Bak activation by an indirect pathway. Furthermore, Bfl-1-α5 can also be distinguished from the homologous segment on Bcl-xL, since a peptide corresponding to this latter helix was not able to induce mitochondrial permeabilization (in spite of a good ability to insert within lipid membranes) [16], [21]. Overall, these findings imply that subtle yet important differences have emerged during evolution not only between homologous membrane-active helices of anti- and pro-apoptotic members but also within the anti-apoptotic sub-group.

The sharpest difference between the homologous membrane-active regions under study comes from the intriguing behavior of Bfl-1-α9 that differs from all the other helices. Indeed, we observed that Bfl-1-α9 induced the release of cytochrome c at a concentration as low as 0.5 µM (i.e. at lower concentrations than the other Bcl-2-like-derived peptides used in our studies). Additionally, we observed the release of large proteins located in the mitochondrial matrix at higher doses, in a Bax/Bak independent manner. Such a membrane permeabilizing activity, including the concentration dependence, is reminiscent of the action of cationic amphipathic antimicrobial peptides [43], [44], [45]. It also resembles the action of Bax-α5 and Bax-α6 peptides on synthetic lipid membranes, for which larger pores at increasing peptide-to-lipid ratio have been described [15]. At least for the cases of Bax-α5 [20] and the antimicrobial peptide magainin [46] it has been shown in experiments with giant unilamelar vesicles that pores are large upon the initial stage of peptide binding to membranes. However, after some time the pores equilibrate to a stabilized, reduced size. This kinetic trace of pore formation might be modified depending on peptide concentration to allow the release of larger molecules at increasing amounts of peptide bound to membranes. An alternative explanation might be a detergent- like (or solubilizing) action, exerted specifically at high peptide concentrations and by means of which big proteins are released after extensive breaking of the membrane [36], [47]. However, we consider this unlikely, since the integral membrane protein Hk1 was never observed in the supernatant of mitochondrial permeabilization assays. Membrane-destabilizing properties for Bfl-1-α9 have already been hinted upon by previous reports, and might be associated with the peculiar distribution of charged residues in the C-terminal end of Bfl-1 that confers a strong amphipathic character compared to the typical hydrophobic stretch (TM) found in other Bcl-2 proteins [48], [49]. Interestingly, canonical Bcl-2-like TM domains and Bfl-1-α9 were postulated to have likely independent evolutionary origins [48]. On the other hand, the pro-apoptotic activity of such peptide, acting directly on the lipid membrane and regardless the expression of Bax or Bak, suggests that it could be used to design a therapeutic strategy to kill a number of tumor cells in which Bax or Bak has been inactivated (see also below).Similar to Bfl-1-α5, Bax-α9 was found to promote cytochrome c release from purified mitochondria in a Bax/Bak-dependent manner. It is possible that this peptide competes with the α9 helix of full-length Bax that is engaged in its BH1-3 hydrophobic cleft [50], leading to activation of the endogenous Bax protein by conformational change. However, other factors may prevent MOM permeabilization within cells, since this region was unable to induce cell death when expressed as a GFP fusion polypeptide.

Aside from evolutionary and mechanistic considerations, the interest of the reductionist strategy used here also lies in the identification of minimal motifs able to disrupt the mitochondrial integrity, which may be used to induce apoptosis in cells such as cancerous cells. To date, our work identified four different candidate peptides for further studies as potential therapeutic agents: Bfl-1-α5, Bfl-1-α9 (this study and [48]), Bax-α5 [41] and Bax-α6 [16], [41]. Of note, the synthetic Bax-α5 peptide was found to trigger (when fused to a cell-penetrating peptide) caspase-dependent apoptosis of cancer cells *in vitro* and *in vivo* [41], demonstrating both the feasibility and effectiveness of this approach. However, such biologically active peptides have to be exogenously administered and should therefore be conjugated to a homing device to achieve selective tumor targeting. An interesting alternative would be to activate the proteolytic cleavage of Bax or Bfl-1 specifically in tumor cells. In that respect, we demonstrated here that µ-calpain is able to cleave Bfl-1 in its N-terminal part *in vitro* within the same region as previously observed *in cellulo* [28]. In both cases, µ-calpain truncation of Bfl-1 leads to the generation of a death fragment that comprises the cytotoxic α5 and α9 helices but is devoid of the BH3 domain, contrary to the other prosurvival Bcl-2 proteins (Bcl-2, Bcl-xL and Mcl-1), for which caspase cleavage produces a pro-apoptotic fragment whose death activity mainly depends on the integrity of the BH3 domain [23], [25], [51]. Although further studies are warranted *in cellulo* to determine whether the cleavage at the amino-acid 71 in Bfl-1 is the major µ-calpain site these results suggest that calpain-truncated Bfl-1 may promote apoptosis through α5/α9-mediated MOM permeabilization, and not as a BH3-only molecule [35]. Because Bfl-1 is endogenously overexpressed in many tumor cells, including chemoresistant Diffuse Large B-cell lymphoma, Hogdkin’s Reed Stenberg cells and melanoma cells [52], [53], [54], [55], [56], it would be interesting to design molecules which can activate µ-calpain specifically in those cancer cells in order to convert Bfl-1 from an anti-apoptotic protein to a mitochondria-permeabilizing factor able to trigger cancer cell suicide.

## Materials and Methods

### Peptides and Drugs

The different peptides used for this study were prepared by solid-phase synthesis as reported [14] in an Applied Biosystems ABI 433A Peptide synthesizer (Foster City, CA, USA) using Fmoc chemistry and Tentagel S-RAM resin (Rapp Polymere, Tübingen, Germany; 0.24 mEq/g substitution) as a solid support. Peptides were purified using a C18 semi-preparative reversed-phase column (Merck, Darmstadt, Germany) by HPLC, to a >95% purity, and their identity was confirmed by Mass Spectrometry. Peptide concentrations were determined from UV spectra using a Jasco spectrophotometer (Jasco, Tokyo, Japan). The amino acid sequences of the peptides are shown in Table S2. Recombinant TNFα (Sigma) was used at 10 ng/ml and cycloheximide (Sigma) at 10 µg/ml. Pre-treatment with the calpain inhibitor ALLN (calbiochem) was performed during 30 min at 20 µM.

### Antibodies

Primary antibodies were as follows: mouse monoclonal Anti-mitochondrial-HSP70 (Abcam), polyclonal anti-Bfl-1 (Abcam), anti-GFP mouse monoclonal antibody (Roche), anti-cleaved caspase-3 rabbit polyclonal antibody (Cell Signaling Technology), anti-α-tubulin (Santa Cruz Biotechnologies), anti-actin (Sigma), anti-cytochrome c antibody (Pharmingen), anti-HK1 (Santa Cruz Biotechnologies), anti-MnSOD (Stressgen). HRP-conjugated goat anti-mouse and goat anti-rabbit secondary antibodies (Roche) were used as secondary antibodies. Western Blot analysis was performed according to standard procedures. SuperSignal West Femto Chemiluminescent Substrate (Pierce) was used for detection of endogenous Bfl-1.

### Cell Culture

HT1080 cells were obtained from the European Collection of Cell Cultures, MEF and MEF DKO (Bax/Bak −/−) mouse embryonic fibroblasts were obtained from Dr. Douglas Green’s laboratory (St Jude Children’s research Hospital) and were cultured as previously reported [57]. iBMK W2, iBMK D3 (Bax/Bak −/−) baby mouse kidney cells were cultured as previously described [58]. BJAB cells were obtained from the cell collection facility of the UMS3444/US8 (CelluloNet Lyon, France) and cultured in RPMI supplemented with 10% FBS. For transient transfection, cells were seeded at a density of 10^5^ cells per 35 mm plate and allowed to grow for 24 h before transfection with plasmids using the Lipofectamine2000 (Invitrogen) according to the manufacturer’s recommendation. For each transfection 3 µg of plasmid DNA was used.

### In vitro Cleavage Assays of Recombinant GST-Bfl-1 and Mapping of the Bfl-1 Cleavage Sites

Recombinant GST-Bfl-1(1–172) or GST-Bfl-1(1–151) were produced in bacteria as previously described [49]. For mapping Bfl-1 cleavage sites, 6 µg of recombinant protein were incubated for 60 min at 30°C with 7 µl of porcine µ-calpain (Calbiochem) in 40 µl of µ-calpain buffer (30 mM TrisHcl pH 7.5, 750 µM CaCl2, 1.5 mM DTT). The different reactions were run on 4–12% NuPAGE Novex (Invitrogen) with MES buffer according to the manufacturer protocol. For digestion of ectopically expressed GFP-tagged Bfl-1 proteins, the constructs were transfected in 293T cells using Lipofectamine 2000 (Invitrogene) and cells were lysed in RIPA buffer without EDTA but supplemented with serine protease inhibitor AEBSF (1 mM). Lysates containing equivalent amount of GFP-Bfl-1 fusion proteins were incubated in calpain buffer in the presence or absence of μ-calpain (0.2 u) for 45 min at 30°C. Reaction was stopped with 1 µl of EDTA (0.5 M).

Samples (gel pieces) were reduced with 60 µL of 10 mM DTT in 50 mM NH_4_HCO_3_ for 15 min at 50°C. Alkylation was performed with 60 µL of 55 mM iodoacetamide in 50 mM NH_4_HCO_3_ for 15 min at room temperature in the dark. The gel pieces were dried using 200 µL of CH_3_CN, protein-containing gel pieces were treated with 0.3–0.5 µg of trypsin (sequence grade, Promega) for 45 min at 50°C. A second extraction step was performed using 30 µL of a H_2_O/CH_3_CN/HCOOH (30/68/2; v/v/v) mixture for 30 min at 30°C, and finally all extracts were pooled and dried in a vacuum concentrator and resuspended in 0.1% trifluoroacetic acid (15 µl).

Mass spectrometry was performed using a Q-Star XL nanoESI Quadrupole/time-of-flight tandem mass spectrometrer, nanoESI-qQ-TOF-MS/MS (Applied Biosystems), coupled to an online nanoliquid chromatography system (Famos, Switchos, and Ultimate from Dionex). The chromatographic separation of peptides was performed in a C18 PepMap micro-precolumn (5 µm; 100 Å; 300 µm×5 mm; Dionex) and a C18 PepMap nano-column (3 µm; 100 Å; 75 µm×150 mm; Dionex). After injection (1 µL injection volume, pick-up mode, in a 15 µL injection loop), samples were adsorbed and desalted on the pre-column with a H_2_O/CH_3_CN/trifluoroacetic acid (98/2/0.05; v/v/v) solvent mixture for 3 min at 25 µL/min flow rate. The peptide separation was developed using a linear 30 min gradient from 0 to 60% B, where solvent A was 0.1% HCOOH in H_2_O/CH_3_CN (95/5) and solvent B was 0.1% HCOOH in H_2_O/CH_3_CN (20/80) at ∼200 nL/min flow rate. MS data were acquired automatically using Analyst QS 1.1 software (Applied Biosystems). The MS and MS/MS data were recalibrated using internal reference ions from a trypsin autolysis peptide at m/z 842.510 [M + H]^+^ and m/z 421.759 [M +2H]^2+^. Screening was achieved by the paragon method from the Protein-Pilot® database-searching software (Applied Biosystems).

To determine the µ-calpain-induced site, 70 µg of GST-Bfl-1(1–151) were digested in 160 µl of reaction with 40 µl of recombinant µ-calpain for 4 hours. Edman degradation of samples was performed on a Procise-492 sequencer (Applied Biosystems, Foster City, Cal, USA) using standard PVDF cycles conditions. Analysis and identification were done through in-line microbore reversed-phase chromatography (140 C Microgradient System, Applied Biosystems, Foster City, Cal, USA), UV detection @ 269 nm, integration and calculation with 610A software (Applied Biosystems, Foster City, Cal, USA).

### Molecular Cloning

The oligonucleotides (Sigma-Proligo) that were used to prepare the different constructions are indicated in Table S1. A double PCR method was used to generate Bfl-1DD and Bfl-1SD mutants using the primers listed in Table S1. All constructions were subcloned into pGEM-T Easy (Promega) and subsequently into XhoI and KpnI sites of pEGFP-C1. The sequence of all constructs was verified by automated sequencing (GEXbyWeb).

### Confocal Microscopy Analysis

Cells were fixed in 4% paraformaldehyde, permeabilized in 0.1% Triton X-100 for 3 minutes, and treated with TO-PRO-3 iodide (final 2 µM, Molecular Probes) before mounting in a drop of anti-bleaching medium. Confocal analysis was performed on a Zeiss confocal microscope (LSM510) (LePecq, France) with a plan apochromat 63×1.4 oil immersion objective. Images were collected under identical non-saturated conditions after multiple scans (∼ 8 sections per cell).

### Measurement of Cell Death and Viability

Hoechst/PI labeling of cells to detect apoptotic and necrotic cell death were performed as described previously [59]. Hoechst 33342 and PI were from Molecular Probes (Invitrogen). Cytotoxicity assays were performed in triplicates. Cell death was quantified by Annexin-V-Cy3 (BioVision Inc.) staining according to manufacturer's protocols, followed by flow cytometric analysis using a FACScan (Becton Dickinson). Data were processed using the CellQuest Pro (version 4.0) software.

### Cytochrome C Release Assays

Purified mitochondria were prepared from iBMK W2, iBMK D3, MEF WT or MEF DKO cells. Briefly, cells were mechanically broken using a 2 ml glass/glass dounce homogenizer (Kontes). Two rounds of 60 and 10 strokes were used for iBMK cells and two rounds of 30 strokes were used for MEF cells. Homogenates were cleared in MB buffer (10 mM Hepes pH 7.5, mannitol 210 mM, sucrose 70 mM, and EDTA 1 mM) at 1500 g and the mitochondria were spun down at 10,500 g. For cytochrome c release assays, 20 µg of purified mitochondria were resuspended in RB buffer (125 mM Kcl, 5 mM succinate and 0.5 mM EGTA) supplemented with protease inhibitor cocktail (Roche). Peptides at different final concentrations were added to the samples and the incubations were carried out at 30°C under agitation (300 rpm). At the indicated time points, samples were spun down at 10,500 g for 5 min at 4°C, supernatants and pellets were recovered and analyzed by immunoblotting for the different markers.

## Supporting Information

Figure S1 **SDS-PAGE patterns of products from µ-calpain-treated GST-Bfl-1(1–151) and GST-Bfl-1(1–172).** Recombinant full length Bfl-1(1–172) and C-terminal truncated Bfl-1(1–151) were treated with µ-calpain *in vitro* and cleaved products were separated by SDS-PAGE. Predominant C-terminal truncated product due to cleavage at F71/N72 site is detected (black arrow) and shifts when full length Bfl-1 protein is digested (white arrow). Cleaved products were confirmed by MS/MS.
(TIF)

Figure S2 **Identification of the Bfl-1 homologous sequence in BCL2L10 overlapping the first μ-calpain site.** Alignment of Bfl-1 and Bcl2L10 N-terminal primary sequences. Homologous sequences in Bcl2L10 surrounding the first cleavage site of Bfl-1 was determined (Box) to design Bfl-1 swapped mutant (Bfl-1SD). The disordered strech following alpha 1 helix of Bfl-1 is indicated with an asterisque.
(TIF)

Figure S3 **µ-calpain resistant mutants of Bfl-1 have a toxic effect when expressed in Hela cells.** Top panels: Hela cells were transfected with GFP-tagged Bfl-1 constructs (wt, DD or SD) and fluorescence was observed using an inverted microscope 20 hours post transfection. Images are representative of the total field. Middle panels: quantification by FACS of the GFP-expressing Hela cells 20 hours post transfection. Results shown are representative of three independent experiments. Bottom panel: FACS assays of Annexin V staining in Hela cells. Transfected cells were stained for phosphatidylserine exposure using Cy3-conjugated Annexin V and the percentage of apoptotic GFP-expressing cells was determined by FACS. Assays were performed in triplicate and a graph representative of the experiment is shown.
(TIF)

Figure S4 **Ectopic expression GFP-tagged C-terminal fragments of Bax and Bfl-1 induces cell death in wt and DKO MEF cells.** Graphs showing cell death (bottom panel) measured by Annexin-V staining of MEF and MEF-DKO cells expressing the different GFP-tagged constructs described in the upper panel. GFP-tranfected cells treated with staurosporine (STS) or left untreated were used as controls.
(TIF)

Table S1 **Sequence of oligonucleotide primers used for this study.**
(TIF)

Table S2 **Sequence of synthetic peptides used for this study.**
(TIF)
