# Supplementary figures and images for: µ-Calpain Conversion of Antiapoptotic Bfl-1 (BCL2A1) into a Prodeath Factor Reveals Two Distinct alpha-Helices Inducing Mitochondria-Mediated Apoptosis
Source: PLoS One. 2012 Jun 20;7(6):e38620. doi: 10.1371/journal.pone.0038620 (PMC3379997; doi:10.1371/journal.pone.0038620)

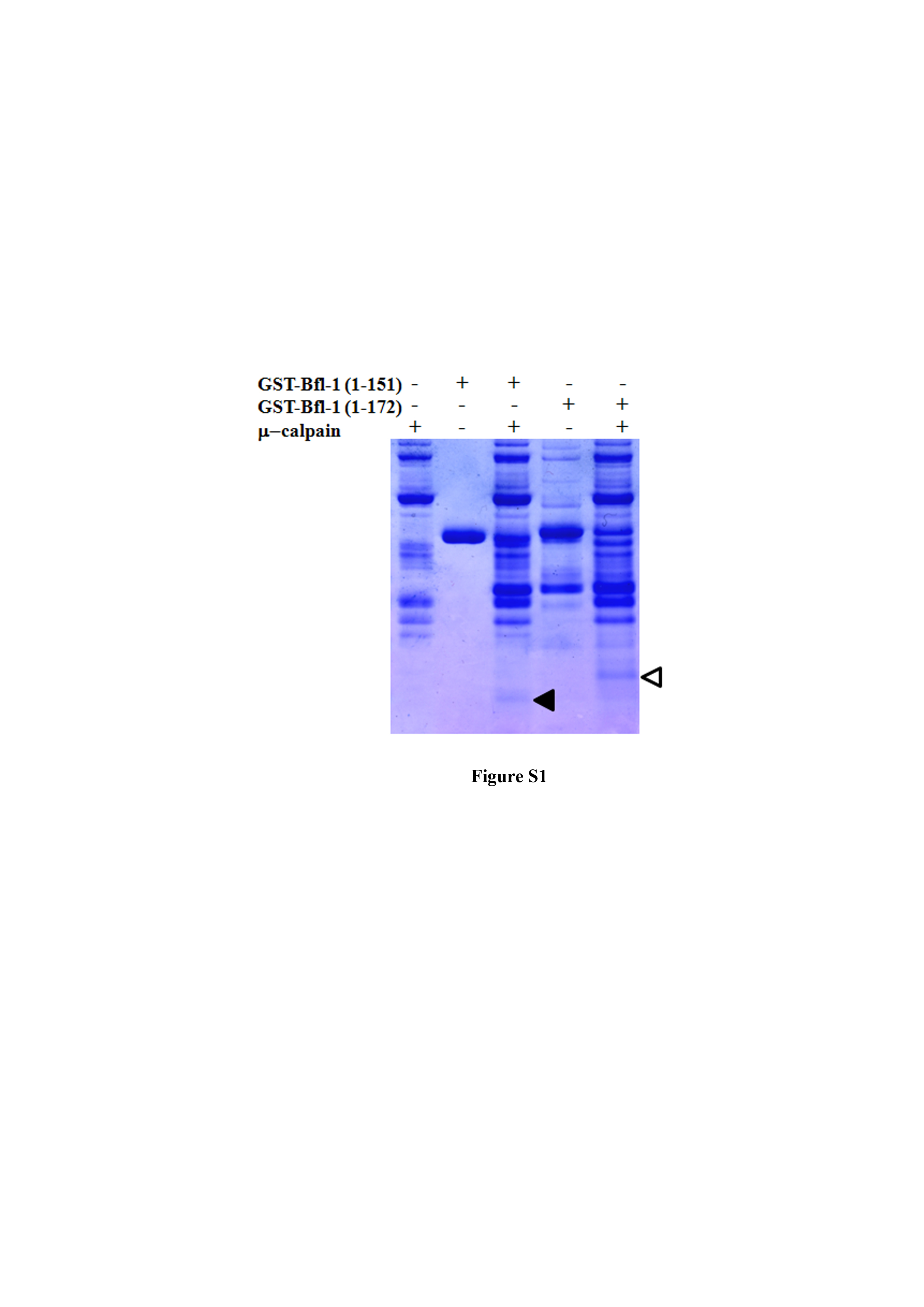

Supplement: Figure S1 — SDS-PAGE patterns of products from µ-calpain-treated GST-Bfl-1(1–151) and GST-Bfl-1(1–172). Recombinant full length Bfl-1(1–172) and C-terminal truncated Bfl-1(1–151) were treated with µ-calpain in vitro and cleaved products were separated by SDS-PAGE. Predominant C-terminal truncated product due to cleavage at F71/N72 site is detected (black arrow) and shifts when full length Bfl-1 protein is digested (white arrow). Cleaved products were confirmed by MS/MS. (TIF) [file pone.0038620.s001.tif]

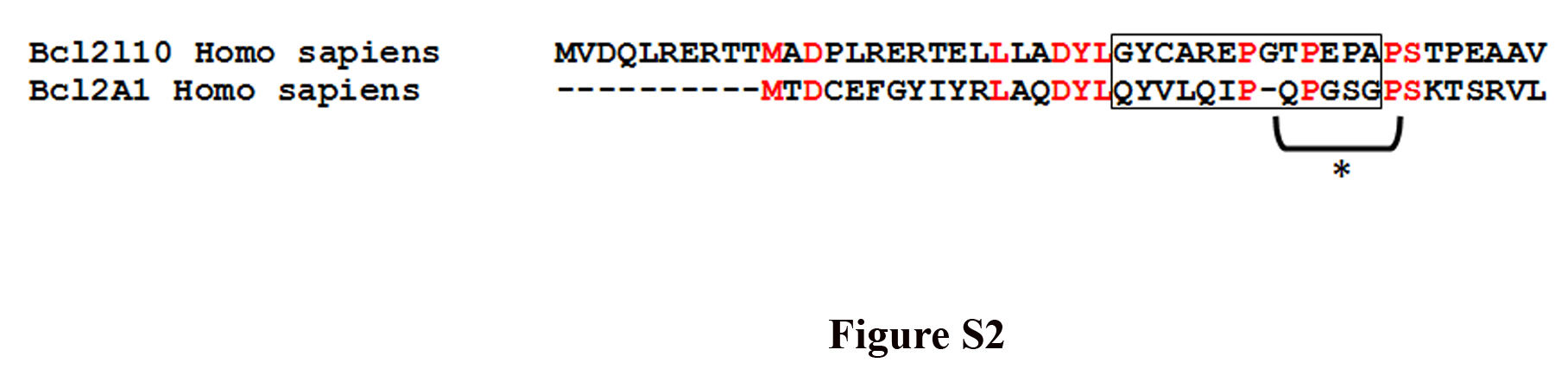

Supplement: Figure S2 — Identification of the Bfl-1 homologous sequence in BCL2L10 overlapping the first μ-calpain site. Alignment of Bfl-1 and Bcl2L10 N-terminal primary sequences. Homologous sequences in Bcl2L10 surrounding the first cleavage site of Bfl-1 was determined (Box) to design Bfl-1 swapped mutant (Bfl-1SD). The disordered strech following alpha 1 helix of Bfl-1 is indicated with an asterisque. (TIF) [file pone.0038620.s002.tif]

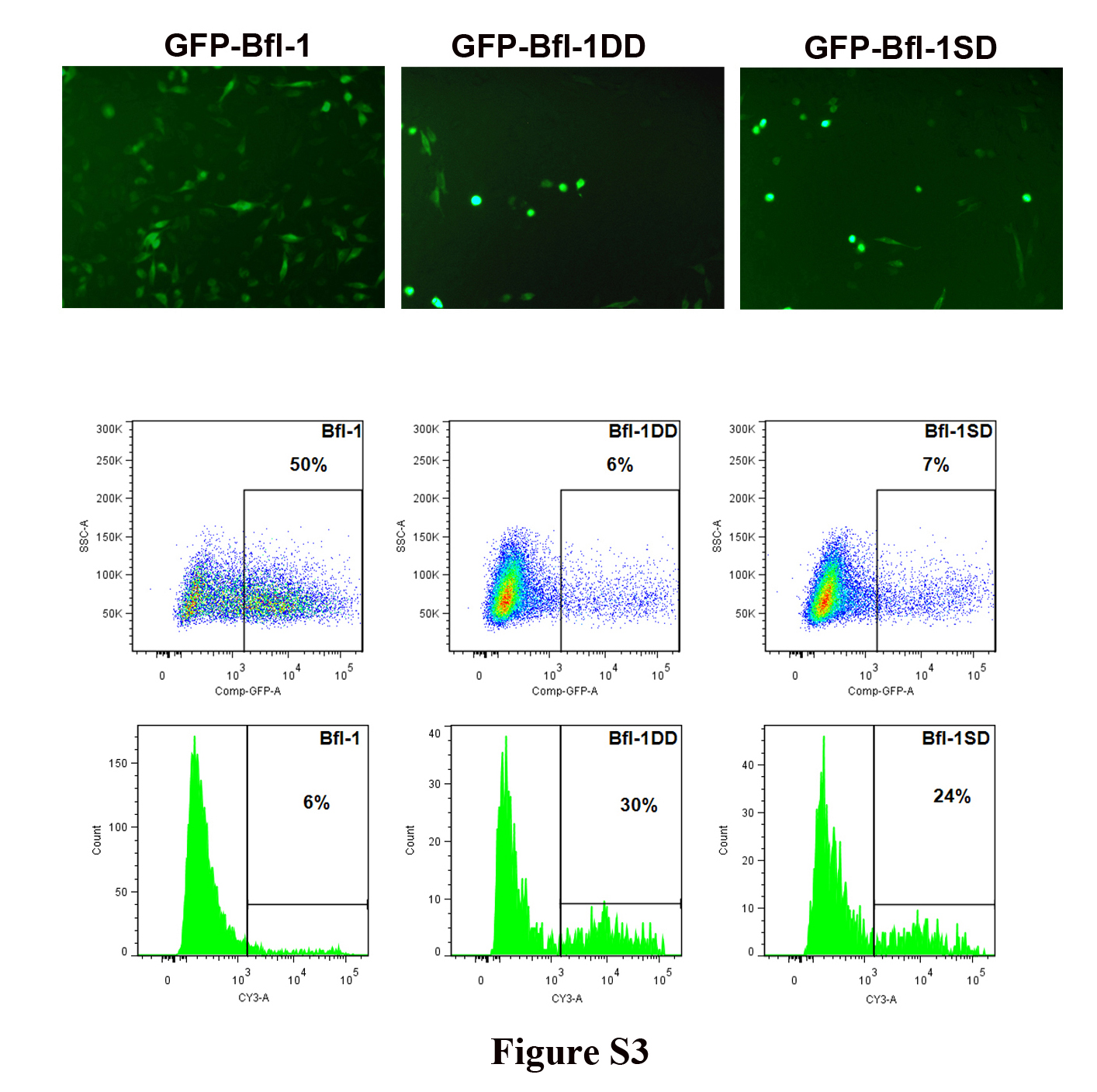

Supplement: Figure S3 — µ-calpain resistant mutants of Bfl-1 have a toxic effect when expressed in Hela cells. Top panels: Hela cells were transfected with GFP-tagged Bfl-1 constructs (wt, DD or SD) and fluorescence was observed using an inverted microscope 20 hours post transfection. Images are representative of the total field. Middle panels: quantification by FACS of the GFP-expressing Hela cells 20 hours post transfection. Results shown are representative of three independent experiments. Bottom panel: FACS assays of Annexin V staining in Hela cells. Transfected cells were stained for phosphatidylserine exposure using Cy3-conjugated Annexin V and the percentage of apoptotic GFP-expressing cells was determined by FACS. Assays were performed in triplicate and a graph representative of the experiment is shown. (TIF) [file pone.0038620.s003.tif]

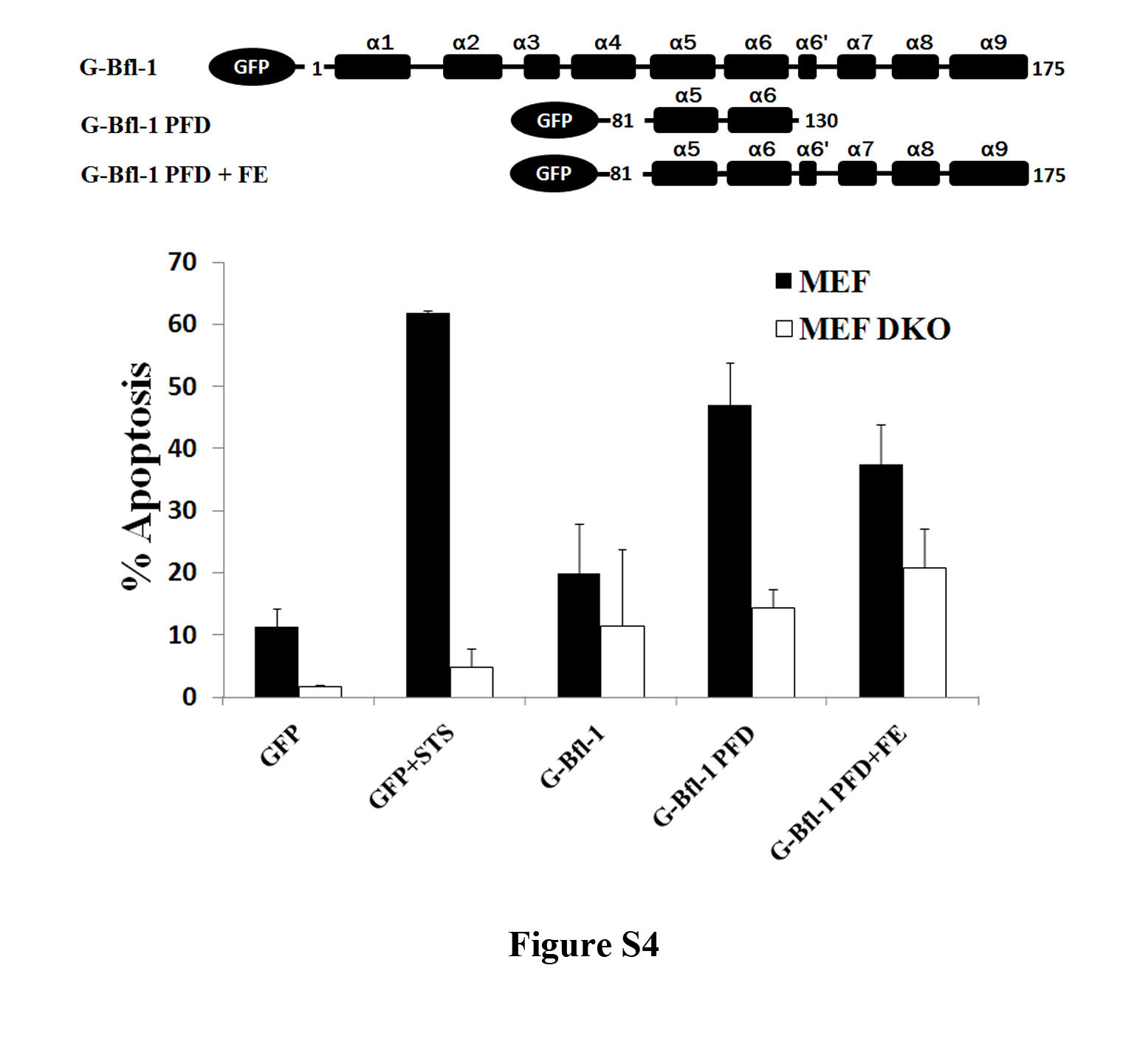

Supplement: Figure S4 — Ectopic expression GFP-tagged C-terminal fragments of Bax and Bfl-1 induces cell death in wt and DKO MEF cells. Graphs showing cell death (bottom panel) measured by Annexin-V staining of MEF and MEF-DKO cells expressing the different GFP-tagged constructs described in the upper panel. GFP-tranfected cells treated with staurosporine (STS) or left untreated were used as controls. (TIF) [file pone.0038620.s004.tif]

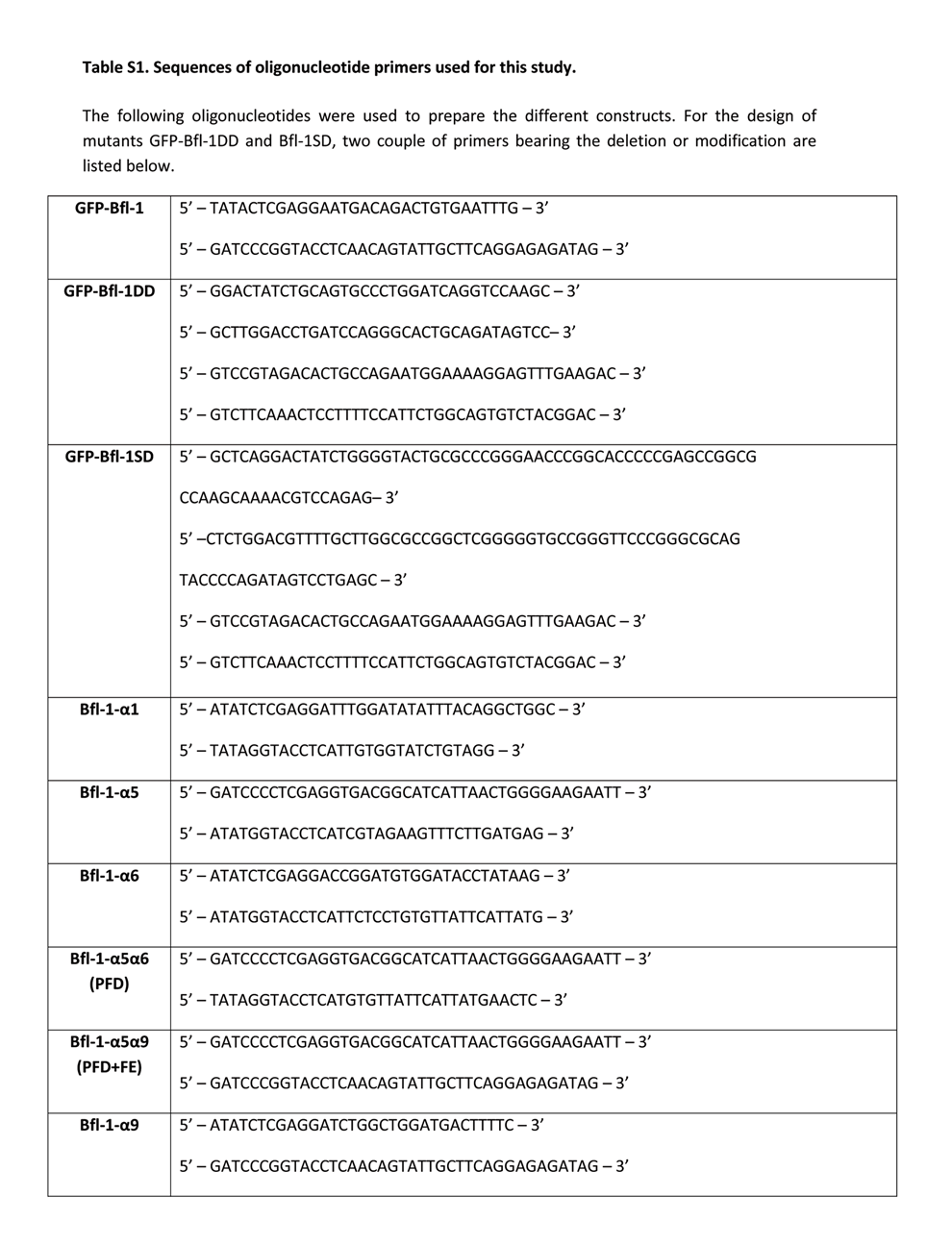

Supplement: Table S1 — Sequence of oligonucleotide primers used for this study. (TIF) [file pone.0038620.s005.tif]

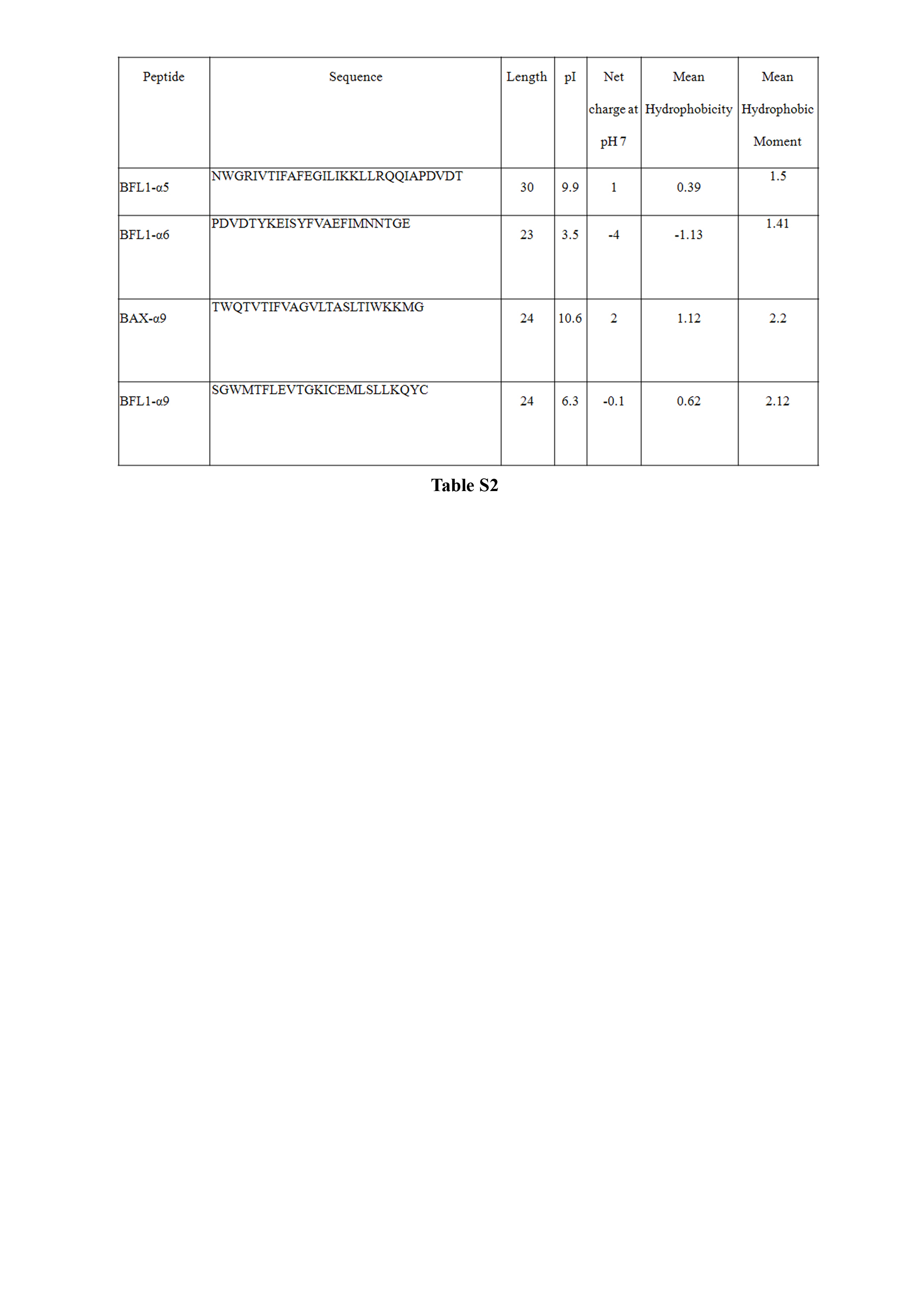

Supplement: Table S2 — Sequence of synthetic peptides used for this study. (TIF) [file pone.0038620.s006.tif]
